# The Gastrointestinal Microbiome: Alcohol Effects on the Composition of Intestinal Microbiota

**DOI:** 10.35946/arcr.v37.2.07

**Published:** 2015

**Authors:** Phillip A. Engen, Stefan J. Green, Robin M. Voigt, Christopher B. Forsyth, Ali Keshavarzian

**Affiliations:** Phillip A. Engen is a researcher in the Department of Internal Medicine, Division of Gastroenterology; Christopher B. Forsyth, Ph.D., is an associate professor in the Department of Internal Medicine, Division of Gastroenterology, and a professor in the Department of Biochemistry; Robin M. Voigt, Ph.D., is an assistant professor in the Department of Internal Medicine, Division of Gastroenterology; all at the Rush University Medical Center, Chicago, Illinois. Stefan J. Green, Ph.D., is director of the DNA Services Facility, Research Resources Center, and is an affiliate professor at the Department of Biological Sciences, University of Illinois at Chicago, Chicago, Illinois. Ali Keshavarzian, M.D., is a director in the Department of Internal Medicine, Division of Gastroenterology, and a professor in the Department of Pharmacology and the Department of Physiology, Rush University Medical Center, Chicago, Illinois; and a professor at the Division of Pharmacology, Utrecht Institute for Pharmaceutical Sciences, Utrecht University, Utrecht, the Netherlands.

**Keywords:** Alcohol consumption, alcohol use, abuse, and dependence, alcohol use disorder (AUD), alcoholic liver disease (ALD), microbiota, intestinal microbiota, microbiota analyses, gastrointestinal microbiome, dysbiosis, probiotics, synbiotics

## Abstract

The excessive use of alcohol is a global problem causing many adverse pathological health effects and a significant financial health care burden. This review addresses the effect of alcohol consumption on the microbiota in the gastrointestinal tract (GIT). Although data are limited in humans, studies highlight the importance of changes in the intestinal microbiota in alcohol-related disorders. Alcohol-induced changes in the GIT microbiota composition and metabolic function may contribute to the well-established link between alcohol-induced oxidative stress, intestinal hyperpermeability to luminal bacterial products, and the subsequent development of alcoholic liver disease (ALD), as well as other diseases. In addition, clinical and preclinical data suggest that alcohol-related disorders are associated with quantitative and qualitative dysbiotic changes in the intestinal microbiota and may be associated with increased GIT inflammation, intestinal hyperpermeability resulting in endotoxemia, systemic inflammation, and tissue damage/organ pathologies including ALD. Thus, gut-directed interventions, such as probiotic and synbiotic modulation of the intestinal microbiota, should be considered and evaluated for prevention and treatment of alcohol-associated pathologies.

It has been estimated that approximately 2 billion people worldwide drink alcohol on a daily basis, with more than 70 million people having a diagnosed alcohol use disorder (World Health Organization 2004). Globally, alcohol use is the fifth leading risk factor for premature death and disability among people between the ages of 15 and 49 (Lim et al. 2012). Excessive alcohol consumption in the United States accounts for 80,000 deaths yearly (Centers for Disease Control and Prevention 2004) and is the third leading preventable cause of death in the United States (Mokdad et al. 2004). In addition, the Centers for Disease Control and Prevention (CDC) found that in 2006, excessive drinking cost the United States more than $224 billion (Bouchery et al. 2011). In a subgroup of alcoholics, alcohol consumption is linked with tissue injury and organ dysfunction, including alcoholic liver disease (ALD) (Purohit et al. 2008), increased risk of developing cancer (Seitz and Stickel 2007), abnormal function of the immune system that increases the risk of acute and chronic infections (Szabo and Mandrekar 2009), pancreatitis (Chowdhury and Gupta 2006), heart disease (Liedtke and DeMuth 1975), and disruption of the circadian clock (Spanagel et al. 2005). The observation that only some alcoholics develop alcohol-induced pathology indicates that, although alcohol is necessary, it is not sufficient to cause organ dysfunction. Consequently, factors other than the toxicity of alcohol are involved in generating health complications, one of which may be alcohol-induced changes in intestinal microbiota composition and/or function.

The intestinal microbiota is classified as the total collection of microbial organisms (bacteria and microbes) within the gastrointestinal tract (GIT). It contains tens of trillions of microorganisms, including at least 1,000 different species of known bacteria, the vast majority of which belong to the phyla *Firmicutes* and *Bacteroidetes* (Ley et al. 2008). The metagenome is the collection of all the different genes found within the gut microbiome; the GIT microbiome contains more than 3 million unique genes, outnumbering the number of human genes 150 to 1 (Proctor 2011). The GIT and the intestinal microbiota display a symbiotic relationship. The microbiota contributes to the extraction of energy from food and synthesis of vitamins and amino acids, and helps form barriers against pathogens (Tappenden and Deutsch 2007). Disruption of intestinal microbiota homeostasis—called dysbiosis—has been associated with inflammatory bowel disease (IBD) (Hold et al. 2014), irritable bowel syndrome (IBS) (Kassinen et al. 2007), celiac disease (Nadal et al. 2007), food allergies (Kuvaeva et al. 1984), type 1 diabetes (Wen et al. 2008), type 2 diabetes (Larsen et al. 2010), cancer (Schwabe and Jobin 2013), obesity (Turnbaugh et al. 2006), and cardiovascular disease (Harris et al. 2012). Although it is unclear whether dysbiosis is the cause or the result of these diseases, factors that contribute to the development and progression of many of these diseases are known to influence the GIT microbiota.

Dysbiosis can be caused by environmental factors commonly encountered in Western societies, including diet (David et al. 2014), disruption of circadian rhythms (Voigt et al. 2014), and alcoholic beverage consumption (Mutlu et al. 2009; Yan et al. 2011) (figure 1). It is well-established that diet influences intestinal microbiota composition and diversity (David et al. 2014) (figure 1). Diets high in fat alter intestinal microbiota (Cani et al. 2007), as do “Western” diets, comprising high fat and high sugar (Turnbaugh et al. 2008). The consequence of diets high in fat or sugar may contribute to the development of obesity and liver injury (Frazier et al. 2011), as well as IBD, IBS, celiac disease, type 1 and type 2 diabetes, food allergies, and cardiovascular disease (Brown et al. 2012; Manzel et al. 2014), at least in genetically susceptible individuals. Alcohol is another dietary disruptor of the intestinal microbiota. A limited number of studies have examined the effects of alcohol on the microbiota in rodents (Mutlu et al. 2009; Yan et al. 2011) and humans (Bode et al. 1984; Chen et al. 2011; Mutlu et al. 2012; Queipo-Ortuno et al. 2012). These changes seem to be relevant for alcohol-associated pathologies because interventions known to alter the intestinal microbiota diminish some alcohol-associated pathologies such as liver disease (Bull-Otterson et al. 2013; Liu et al. 2004; Mutlu et al. 2009).

In this review, we examine alcohol-induced effects on microbiota and how interventions targeted at normalizing alcohol-induced dysbiosis may mitigate some of the detrimental effects of alcohol.

## Analyzing the Intestinal Microbial Community

**Figure 2:**
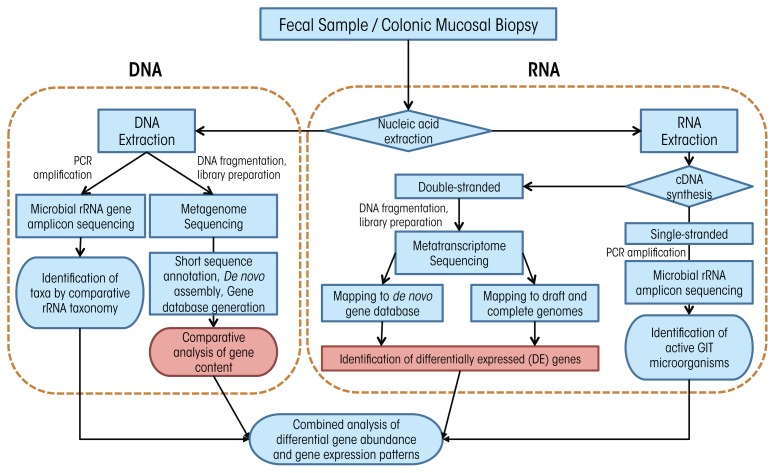
Basic pipeline for amplicon-based and shotgun sequencing approaches to the interrogation of GIT microbial communities. Nucleic acids can be interrogated independently to characterize the community structure and gene content of total (DNA) and active (RNA) microbial communities or combined to examine how shifts in microbiota are correlated with changes in community gene expression patterns.

Before we can understand the influence of alcohol on the GIT microbiota, we need to understand a bit about how researchers measure these microorganisms and evaluate changes in their populations. In fact, it is difficult to directly measure microbial communities such as those within the GIT because of a number of confounding factors. For one, microorganisms maintain incredible genetic diversity but house this diversity in an extraordinarily limited array of cellular morphologies (Woese 1987). In addition, microorganisms have redundant functional capabilities, share divergent functional capabilities with closely related microorganisms, have the potential for high metabolic diversity within single microbial lineages, and are extraordinarily difficult to isolate under laboratory conditions. Taken together, these confounding factors compel researchers to use molecular tools—tools that examine DNA and RNA—to analyze these complex communities. These tools fall into two broad categories: polymerase chain reaction (PCR)–based targeted approaches and shotgun sequencing approaches (figure 2), which we explain in detail in the sidebar.

Because it is exceedingly difficult to obtain microbial samples from different locations in the GIT, researchers overwhelmingly extract the genomic DNA they need to analyze the GIT microbiota from mucosa-associated colonic tissue biopsies and from fecal samples. However, using these samples assumes that the colonic tissue and feces are a suitable proxy for the GIT. A study (Stearns et al. 2011) addressed this issue in an analysis of microbiota community structure in mouth, stomach, duodenum, colon, and stool, via gastroscopy and colonoscopy from four healthy individuals. When examined in the context of the entire GIT, colonic tissue and fecal samples were most similar to each other in all individuals. However, the community composition was substantially altered in colon and fecal samples from the same individual: three of four individuals had a much reduced level of microorganisms from the phylum *Bacteroidetes* in fecal samples. This led to a substantially altered ratio of *Firmicutes* to *Bacteroidetes,* a ratio that has been used as a diagnostic parameter in studies of disease (see sidebar). Eckburg and colleagues (2005) also found a similar divergence between GIT colonic tissue and fecal microbiota. Thus, although colonic tissue and fecal samples will continue to serve as common, imperfect proxies for GIT microbiota, they should not be considered a perfect representation of the entire GIT microbial community, which undergoes dramatic changes from the stomach to colon (Stearns et al. 2011). No obvious solution is available, leaving only highly invasive sampling techniques as a mechanism to collect samples from multiple locations of the GIT.

## Alcohol-Induced Effects and Implications on the Intestinal Microbiota

The study of alcohol’s effects on the structure and activity of GIT microbiota still is in its infancy, particularly compared with other alcohol-induced effects. The literature reviewed below demonstrates that alcohol consumption leads to quantitative and qualitative dysbiosis in the intestinal microbiota of rodents and humans (table 1). These studies demonstrate alterations in the dominant bacterial taxa from the phyla *Bacteroidetes* and *Firmicutes* and, in several studies, an increase in bacteria from the phylum *Proteobacteria*.

### Rodent Models

Studies in mice and rats find both alcohol-induced bacterial overgrowth and dysbiosis. In one study, C57BL/6 mice were intragastrically fed alcohol (30.9 g/kg per day; 40 percent of their total daily calories from alcohol) for 3 weeks and compared with control mice intragastrically fed an isocaloric liquid diet. The alcohol-fed mice developed ALD, which was associated with small intestinal bacterial overgrowth and dysbiosis in the cecum—the beginning of the large intestine (Yan et al. 2011). In particular, the GIT microbiota of alcohol-treated mice showed a decrease in *Firmicutes* and an increase in the relative abundance of *Bacteroidetes* and *Verrucomicrobia*, among other bacteria (table 1). In comparison, the GIT microbiota of control-fed mice showed a relative predominance of bacteria from the phylum *Firmicutes*. In a separate study, Sprague-Dawley rats intragastrically fed alcohol daily (8 g/kg per day) for 10 weeks showed altered colonic mucosa–associated bacterial microbiota composition leading to ileal and colonic dysbiosis (Mutlu et al. 2009). In prior studies, Sprague-Dawley rats developed intestinal oxidative stress, intestinal hyperpermeability, endotoxemia, and steatohepatitis by the 10th week of alcohol treatment (Keshavarzian et al. 2009), suggesting that changes in the microbiota may be contributing to the alcohol-induced effects on the intestine and liver. Intestinal dysbiosis may potentially contribute to the pathogenesis of liver disease by altering intestinal barrier integrity, resulting in intestinal hyperpermeability, as well as increased production of proinflammatory factors that could both promote liver pathology.

### Humans

**Figure 3:**
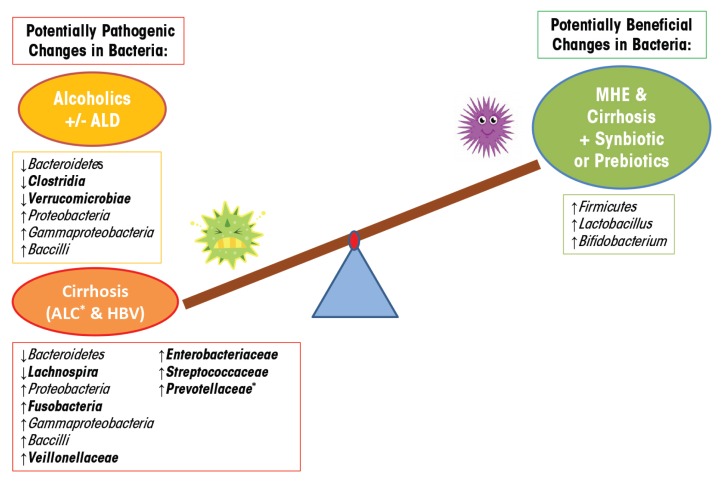
Alcohol-induced imbalances in the microbiome of the gastrointestinal tract (dysbiosis) have been associated with promoting potentially pathogenic changes in bacteria in alcoholics with and without liver disease and in patients with cirrhosis caused by hepatitis B or alcohol. Both alcoholic and cirrhosis patients demonstrate similar dysbiotic microbiota changes, except for the bacteria indicated, suggesting that these dysbiotic bacterial differences could contribute to liver disease or may be a biomarker indicating liver disease. Using synbiotics and prebiotics to treat Minimal Hepatic Encephalopathy patients with cirrhosis, significantly improved their GIT microbiota, suggesting that the same treatment may benefit patients with alcohol-induced dysbiosis.

Chronic alcohol consumption in humans also causes bacterial overgrowth and dysbiosis. One study using culture-based methods, for example, found alcohol-induced alterations, including small intestine bacterial overgrowth of both aerobic and anaerobic bacteria in the jejunum (Bode et al. 1984). Another study showed that alcohol consumption alters the composition of mucosa-associated microbiota in human sigmoid biopsies taken from alcoholics with and without ALD as well as healthy control subjects (Mutlu et al. 2012). In this study, the researchers used 16S rRNA gene sequencing to assess the microbiota. They found that the microbial community was significantly altered—containing a lower abundance of *Bacteroidetes* and a higher abundance of *Proteobacteria—*in a subgroup of alcoholics with and without liver disease (table 1). Other studies show that dysbiotic microbiota in alcoholics also correlates with a high level of endotoxin in the blood, indicating that dysbiosis may contribute to intestinal hyperpermeability and/or the increased translocation of gram-negative microbial bacterial products from the intestinal lumen into systemic circulation (Mutlu et al. 2009; Rimola 1991). Similarly, 16S rRNA gene analysis of fecal microbiota from human subjects with hepatitis B or alcohol-related cirrhosis shows a reduction in *Bacteroidetes* and an increase in *Proteobacteria* and *Fusobacteria*, compared with healthy control subjects (table 1) (Chen et al. 2011). At a finer taxonomic resolution, this study also shows a significant increase in potentially dangerous bacteria from the families *Prevotellaceae*, *Enterobacteriaceae, Veillonellaceae,* and *Streptococcaceae* in subjects with alcoholic cirrhosis, compared with subjects with hepatitis B cirrhosis and with control subjects. The prevalence of potentially pathogenic bacteria in patients with cirrhosis may affect prognosis, something supported by previous research (Guarner et al. 1997; Liu et al. 2004). Other lower resolution studies find that the relative abundance of bacteria from the phylum *Bacteroidetes* decreases as those from the phylum *Proteobacteria* increase and that individuals with cirrhosis exhibit a unique increase in *Fusobacteria* (Chen et al. 2011; Mutlu et al. 2012). Overall, alcoholics and cirrhosis patients demonstrate microbial communities enriched in *Proteobacteria* of the class *Gammaproteobacteria* and *Firmicute* of the class *Bacilli.* In contrast, *Firmicutes* of the class *Clostridia* are depleted in alcoholics but are not significantly changed in the cirrhosis group, with the exception of *Veillonellaceae,* which is increased and *Lachnospira,* which is decreased (table 1). These findings suggest that microbiota community differences between alcoholics and alcoholics with cirrhosis (e.g., *Fusobacteria, Clostridia*) may contribute to the development of liver disease or may be a biomarker indicating liver disease (figure 3). Future studies will need to determine the cause-and-effect relationship of the microbiota community structure and liver disease.

**Methods for Analyzing the Gastrointestinal Microbiota**

To understand the results of microbiota analyses, it can help to understand a bit about the methods researchers use. As mentioned in the main article, researchers tend to use techniques that look for DNA and RNA related to specific microorganisms. To do that, they typically use one of two techniques: polymerase chain reaction (PCR) and shotgun sequencing. Here, we explain in general terms how each method is used to analyze GIT microbiota.

***PCR***

To successfully use PCR, researchers needed to find an appropriate gene target that would be common enough among microorganisms so they could use a known segment for searching but different enough so that they could individuate among microorganisms. They quickly selected ribosomal RNA (rRNA) genes (Pace 1986; Woese 1987). Ribosomal RNAs are essential for protein synthesis within all cells and therefore their genes have many features that make them desirable for determining the makeup of complex microbial communities. In particular, the genes contain regions of DNA that are highly variable among species and so can serve as a kind of identifier; but they also contain regions that are highly conserved, or the same among many species, and are therefore suitable for the development of broad-range PCR primers that use snippets of known DNA to search for specific genes. As a result of these features, rRNA genes have become the “gold standard” for molecular analyses, and they are typically analyzed using PCR-based techniques coupled with indirect fingerprinting or direct sequencing, including with next-generation sequencing (NGS). To profile GIT microbial communities using rRNA gene analysis, researchers typically extract genomic DNA from mucosa-associated colonic biopsies and fecal matter. They then use PCR to amplify the DNA, creating what are called “amplicons,” using primers targeting conserved regions of the small subunit (SSU or 16S) rRNA gene from all bacteria and sometimes archaea. The researchers then sequence these PCR amplicons after suitable preparation for the chosen sequencing platform (Langille et al. 2013). Whereas it was previously common to have clone libraries on the order of 100 sequences per sample, it is more typical with NGS approaches to have sequence libraries of 10,000 to 100,000 sequences per sample. A suite of bioinformatics tools has been developed to process this high-throughput data such as RDP (Cole et al. 2005), mothur (Schloss et al. 2009), and QIIME (Caporaso et al. 2010).

Because of limitations inherent in the analysis of a structural gene, such as the rRNA gene that is common to all organisms, this method should be viewed as the first step in a multi-tiered approach to the analysis of microbial communities. The following are some limitations: (1) rRNA gene sequencing does not provide definitive physiological information about an organism; (2) for DNA-based methods, the presence of an organism’s rRNA gene does not guarantee that the organism is active in the studied system at the time of sampling; (3) variation in the number of rRNA genes among bacterial lineages distorts the true diversity of microorganisms in an environmental sample; and (4) difficulty in species- and strain-level phylogenetic resolution among some taxa, depending upon the region of rRNA gene analyzed. Nonetheless, for large studies with many samples, a preliminary screen using this method is often suitable for identifying large-scale shifts in microbial community structure and for identifying statistically significant changes in the relative abundance of organisms between groups or treatments.

That said, the interpretation of results from the analysis of microbial community composition using DNA-based methods can be confounded by the presence of DNA from dead, dormant, or weakly active organisms contributing little to overall microbial community function. To circumvent these limitations, researchers can directly target rRNAs instead of rRNA genes. In such an approach, researchers extract total RNA from an environmental sample and reverse transcribe this RNA using either a random primer mix or a gene-specific “reverse” primer matching the rRNA (figure 2). This process generates single-stranded complementary DNA (cDNA), which is then used as a template for PCR and sequencing with domain-level primer sets as is done with genomic DNA. As microbial RNA is labile and degrades rapidly if not continually produced, rRNA analysis reflects only active microorganisms, and the relative abundance of rRNAs represents the relative activity of organisms in the system. Although rRNA analysis still does not provide an explicit link to physiology for most organisms, such analyses may find stronger correlation to measured functions at the time of sampling. Microbial RNA degrades rapidly, and for GIT colonic tissue and fecal samples, the time delay until RNA can be extracted may result in a serious distortion of active organisms and gene expression patterns from in situ. Thus, animal model systems in which animals are killed for sampling may be more suitable for RNA studies as mRNAs and ribosomes can be preserved rapidly for downstream analyses.

***Shotgun Metagenomic and Metatranscriptomic Sequencing***

Although amplicon sequencing approaches are extremely useful for GIT microbiota community characterization, they are limited by the need to have some known DNA sequences to look for. Therefore, to detect novel genes and gene variants, it is necessary to have sequencing approaches that do not depend on such information. Researchers use so-called “shotgun” sequencing approaches (figure 2) to circumvent the need for a priori sequence information through the use of molecular manipulations of nucleic acids to attach known sequences for priming of sequencing reactions to unknown sequences. Shotgun sequencing approaches, in which no a priori selection of a region or gene of interest is performed, provides a holistic view of microbial communities, gene content, and expression patterns. However, low-abundance taxa or those with small genomes, like viruses, may be swamped out by high-abundance or large genome organisms and may benefit from targeted amplification approaches.

Two techniques are used for more detailed assessments of GIT microbiota functional capabilities: In shotgun metagenomics, total genomic DNA is fragmented and sequenced directly (Qin et al. 2010), and in shotgun metatranscriptomics, fragmented messenger RNAs are sequenced directly (Perez-Cobas et al. 2013). These techniques can provide data to identify active organisms and metabolic activities at the time of sampling (metatranscriptome) and to directly link community function to specific microbial lineages, even at the species or subspecies level (metagenome and metatranscriptome). Such in-depth analyses can identify key GIT microbiota community members, identify essential genes associated with the GIT microbiota, and improve metabolic modeling to predict the physiology of dominant organisms in environments undergoing global changes (Greenblum et al. 2012; Karlsson et al. 2013; Qin et al. 2010). Metagenome sequencing can provide much more detailed taxonomy of communities based on genes other than rRNAs, particularly at the species and strain level (Morowitz et al. 2011; Poretsky et al. 2014). In particular, GIT microbiota analyses of disease states and obesity have found widespread application (Greenblum et al. 2012; Karlsson et al. 2012, 2013; Manichanh et al. 2006; Qin et al. 2012). A full survey of the methods for analysis of metagenomic data is beyond this review; however, many recent articles provide deeper overviews (Cho and Blaser 2012) and describe suitable pipelines (Huson et al. 2007; Meyer et al. 2008; Treangen et al. 2013; Zakrzewski et al. 2013).

Although powerful, these approaches are limited by many factors:

- High cost attributed to heavy sequence demand;
- Insufficiently robust reference databases to provide suitable annotation to all recovered gene fragments;
- High microbial diversity in the GIT, which leads to limited coverage of most organisms aside from highly abundant organisms;
- High transcript abundance of housekeeping genes; and
- High computer memory and computational demand for analysis.

Because of the relatively high cost of shotgun sequencing approaches relative to amplicon sequencing approaches (typically about 20 to 30 times higher cost), researchers must carefully tailor their project goals to the appropriate molecular methodology. In a tiered sequencing approach, researchers perform amplicon sequencing on all samples and use their analysis of amplicon data to select critical or representative samples for deeper sequence analysis.

**Considerations for Nucleic Acid Extraction**

Analysis of gastrointestinal tract (GIT) microbiota communities presents several features worthy of consideration. In particular, researchers take the majority of samples from feces and mucosa-associated colonic tissue biopsies. Traditionally, extraction of nucleic acids from mammalian feces generated nucleic acid templates of poor purity. However, new extraction protocols and commercial kits have largely removed nucleic acid purity as a limitation to downstream molecular analyses (Claassen et al. 2013; Ó Cuív et al. 2011). Indeed, many manufacturers produce kits specifically for GIT colonic tissue and fecal DNA extraction (e.g., Mo Bio PowerFecal^®^ DNA Isolation Kit; Qiagen QIAamp DNA Stool Mini Kit; Zymo ZR Fecal DNA MiniPrep kit; Epicentre ExtractMaster^™^ Fecal DNA Extraction Kit). Although many of these extraction kits have similar chemistry, other features of the kits may be critical to the maximum recovery of genomic DNA from GIT colonic tissue and feces and to minimize distortion of the GIT microbiota community as a result of differential lysis of different types of microbial cells.

Mammalian GIT microbiota communities are dominated by bacteria from two phyla: *Bacteroidetes* and *Firmicutes* (Ley et al. 2008), and researchers have used the ratio of these phyla as a diagnostic parameter. For example, Mariat and colleagues (2009) observed dramatic age-related changes in the ratio of *Firmicutes* and *Bacteroidetes* (F/B) in feces from healthy individuals, and the ratio has been broadly utilized in studies of obesity, with greater numbers of *Firmicutes* in obese patients (Ley et al. 2006). That said, sampling processing procedures can affect this ratio because the phylum *Firmicutes* consists of mostly gram-positive bacteria with thick cell walls that can make them difficult to lyse, thus high-energy lysis steps (e.g., bead-beating) are important in extraction protocols. In addition, lytic enzymes such as lysozyme, mutanolysin, and lysostaphin can be used individually or in combination to enhance lysis of difficult-to-lyse organisms (Yuan et al. 2012). One study (Bahl et al. 2012) demonstrated that freezing of fecal samples prior to DNA extraction can alter the F/B ratio, with enhanced relative abundance of *Firmicutes* after freezing. As a result of these issues, it may be difficult to easily compare directly between studies of fecal samples processed under different conditions. Likewise, protocols should be carefully considered and rigorously adhered to in order to provide reproducible handling for each sample.

ReferencesBahlMIBergstromALichtTRFreezing fecal samples prior to DNA extraction affects the *Firmicutes* to *Bacteroidetes* ratio determined by downstream quantitative PCR analysisFEMS Microbiology Letters329219319720122232500610.1111/j.1574-6968.2012.02523.xCaporasoJGKuczynskiJStombaughJQIIME allows analysis of high-throughput community sequencing dataNature Methods7533533620102038313110.1038/nmeth.f.303PMC3156573ChoIBlaserMJThe human microbiome: At the interface of health and diseaseNature Reviews Genetics134260270201210.1038/nrg3182PMC341880222411464ClaassenSdu ToitEKabaMA comparison of the efficiency of five different commercial DNA extraction kits for extraction of DNA from faecal samplesJournal of Microbiological Methods94210311020132368499310.1016/j.mimet.2013.05.008PMC5809576ColeJRChaiBFarrisRJThe Ribosomal Database Project (RDP-II): Sequences and tools for high-throughput rRNA analysisNucleic Acids Research33D294D29620051560820010.1093/nar/gki038PMC539992GreenblumSTurnbaughPJBorensteinEMetagenomic systems biology of the human gut microbiome reveals topological shifts associated with obesity and inflammatory bowel diseaseProceedings of the National Academy of Sciences of the United States of America109259459920122218424410.1073/pnas.1116053109PMC3258644HusonDHAuchAFQiJSchusterSCMEGAN analysis of metagenomic dataGenome Research17337738620071725555110.1101/gr.5969107PMC1800929KarlssonFHFakFNookaewISymptomatic atherosclerosis is associated with an altered gut metagenomeNature Communications31245201210.1038/ncomms2266PMC353895423212374KarlssonFHTremaroliVNookaewIGut metagenome in European women with normal, impaired and diabetic glucose controlNature49874529910320132371938010.1038/nature12198LangilleMGZaneveldJCaporasoJGPredictive functional profiling of microbial communities using 16S rRNA marker gene sequencesNature Biotechnology319814821201310.1038/nbt.2676PMC381912123975157LeyREHamadyMLozuponeCEvolution of mammals and their gut microbesScience32058831647165120081849726110.1126/science.1155725PMC2649005LeyRETurnbaughPJKleinSGordonJIMicrobial ecology: Human gut microbes associated with obesityNature44471221022102320061718330910.1038/4441022aManichanhCRigottier-GoisLBonnaudEReduced diversity of faecal microbiota in Crohn’s disease revealed by a metagenomic approachGut55220521120061618892110.1136/gut.2005.073817PMC1856500MariatDFirmesseOLevenezFThe Firmicutes/Bacteroidetes ratio of the human microbiota changes with ageBMC Microbiology912320091950872010.1186/1471-2180-9-123PMC2702274MeyerFPaarmannDD’SouzaMThe metagenomics RAST server: A public resource for the automatic phylogenetic and functional analysis of metagenomesBMC Bioinformatics938620081880384410.1186/1471-2105-9-386PMC2563014MorowitzMJDenefVJCostelloEKStrain-resolved community genomic analysis of gut microbial colonization in a premature infantProceedings of the National Academy of Sciences of the United States of America10831128113320112119109910.1073/pnas.1010992108PMC3024690Ó CuívPAguirre de CarcerDJonesMThe effects from DNA extraction methods on the evaluation of microbial diversity associated with human colonic tissueMicrobial Ecology61235336220112115363410.1007/s00248-010-9771-xPaceNAStahlDALaneDJOlsenGJThe analysis of natural microbial populations by ribosomal RNA sequencesAdvances in Microbial Ecology91551986Perez-CobasAEGosalbesMJFriedrichsAGut microbiota disturbance during antibiotic therapy: A multi-omic approachGut62111591160120132323600910.1136/gutjnl-2012-303184PMC3812899PoretskyRRodriguezRLLuoCStrengths and limitations of 16S rRNA gene amplicon sequencing in revealing temporal microbial community dynamicsPLoS One94e9382720142471415810.1371/journal.pone.0093827PMC3979728QinJLiYCaiZA metagenome-wide association study of gut microbiota in type 2 diabetesNature4907418556020122302312510.1038/nature11450QinJLiRRaesJA human gut microbial gene catalogue established by metagenomic sequencingNature4647285596520102020360310.1038/nature08821PMC3779803SchlossPDWestcottSLRyabinTIntroducing mothur: Open-source, platform-independent, community-supported software for describing and comparing microbial communitiesApplied and Environmental Microbiology75237537734120091980146410.1128/AEM.01541-09PMC2786419TreangenTJKorenSSommerDDMetAMOS: A modular and open source metagenomic assembly and analysis pipelineGenome Biology141R220132332095810.1186/gb-2013-14-1-r2PMC4053804WoeseCRBacterial evolutionMicrobiology Reviews512221271198710.1128/mr.51.2.221-271.1987PMC3731052439888YuanSCohenDBRavelJEvaluation of methods for the extraction and purification of DNA from the human microbiomePLoS One73e3386520122245779610.1371/journal.pone.0033865PMC3311548ZakrzewskiMBekelTAnderCMetaSAMS: A novel software platform for taxonomic classification, functional annotation and comparative analysis of metagenome datasetsJournal of Biotechnology167215616520132302655510.1016/j.jbiotec.2012.09.013

Although alcohol can cause intestinal dysbiosis, some alcoholic beverages contain compounds that may favorably alter the GIT microbiota community composition. A study showed the effects of dietary polyphenols on the human GIT microbiota in human healthy control subjects who consumed red wine (272 mL per day), de-alcoholized red wine (272 mL per day), or gin (100 mL per day) for 20 days and had their total fecal DNA assessed from stool collected at baseline and after treatment (Queipo-Ortuno et al. 2012). Red wine polyphenol significantly increases the abundance of *Proteobacteria*, *Fusobacteria*, *Firmicutes* and *Bacteroidetes*, whereas gin consumption significantly decreases these same bacterial phyla (table 1). De-alcoholized red wine consumption significantly increases *Fusobacteria,* and gin consumption increases *Clostridium* abundance compared with de-alcoholized and red wine (table 1). Red wine and de-alcoholized red wine consumption increases the abundance of *Bifidobacterium*, a bacterium that has been shown to be beneficial in the GIT (Gibson et al. 1995). Thus, it seems that polyphenol consumption is associated with an increase in bacteria that are known to promote GIT health, whereas alcohol consumption alone may be damaging to the microbiota balance. The significant decrease of *Clostridium* associated with the consumption of red wine polyphenols suggests that polyphenols may have an inhibitory effect on the growth of *Clostridium,* which has been linked to the progression of colonic cancer and the onset of IBD (Guarner and Malagelada 2003). These results indicate that polyphenol consumption may be used as a dietary intervention to alter the microbiota in a specific way. In addition, daily moderate consumption of red wine polyphenols increases the growth of *Bifidobacterium*, which could be associated with positive prebiotic effects of GIT microbiota, production of beneficial organic acids, and the growth inhibition of pathogenic bacteria (Gibson et al. 1995). Also, as an important consideration to evaluating alcohol-induced effects on the GIT microbiota, differences attributed to the type of alcohol consumption may be contributing to intra- and interstudy variability.

Whether alcohol-induced dysbiosis contributes to the pathogenesis of diseases, such as ALD or alcohol-related cirrhosis, is undetermined. Future studies will need to determine the biological, functional, and clinical significance of the dysbiotic intestinal microbiota composition in alcohol-related disorders.

## From Dysbiosis to Disease

Once alcohol disrupts the intestinal microbiota, both the microbiota and microbiome may increase susceptibility to pathological changes (Lozupone et al. 2012). The majority of the reviewed studies indicate an association between alcohol-induced intestinal bacterial overgrowth and dysbiosis and the development/progression of ALD and cirrhosis. Indeed, disrupted intestinal barrier function, which is associated with alcohol consumption, in combination with alcohol-induced bacterial overgrowth and dysbiosis, could be highly relevant for the development of alcohol-induced liver pathology, including nonalcoholic fatty liver disease (NAFLD), nonalcoholic steatohepatitis (NASH), and ALD. Studies show that alcohol consumption disrupts the intestinal barrier (Keshavarzian et al. 1999) via increasing oxidative stress burden in the intestine, which in turn disrupts tight junctions and promotes intestinal hyperpermeability (Rao et al. 2004). Increased intestinal hyperpermeability allows proinflammatory/pathogenic microbial products, including endotoxin (e.g., lipopolysaccharide [LPS] and peptidoglycan), to translocate from the intestinal lumen to the liver via the portal vein (Frazier et al. 2011). Exposure to these bacterial products causes inflammation in the liver, which may work in conjunction with the direct effects of alcohol to cause ALD (Schnabl and Brenner 2014). This translocation of viable bacterial products during bacterial overgrowth or alcohol-induced dysbiosis may significantly contribute to end-stage liver disease observed in alcohol cirrhosis patients and may therefore contribute to the mortality of cirrhotic patients by inducing infection (Schnabl and Brenner 2014).

## Interventions to Normalize Alcohol-Induced Intestinal Dysbiosis

Research in rodents and humans has begun to investigate whether alcohol-induced intestinal dysbiosis and its consequences may be reversible with probiotic and synbiotic interventions (table 2). Probiotics are live microorganisms that, when taken by the host, have beneficial effects on the host beyond their simple nutritive value (Ewaschuk and Dieleman 2006). Synbiotics are a combination of probiotics and prebiotics—nondigestible fibrous compounds, such as oats, that stimulate the growth and activity of advantageous bacteria in the large intestine.

Probiotics, especially *Lactobacillus rhamnosus* GG (LGG), have several beneficial effects on intestinal function, including stimulating intestinal development and mucosal immunity, ameliorating diarrhea, prolonging remission in ulcerative colitis and pouchitits, reducing intestinal oxidative stress, and maintaining or improving intestinal barrier function (Bruzzese et al. 2004; Ewaschuk and Dieleman 2006; O’Hara and Shanahan 2006; Resta-Lenert and Barrett 2003; Sartor 2004; Tao et al. 2006; Versalovic 2007). Synbiotics have been demonstrated to favorably alter liver metabolism in alcohol-fed animals (Martin et al. 2009).

Studies in rodents demonstrate that both probiotics and prebiotics prevent alcohol-induced dysbiosis. A study in Sprague-Dawley rats that had consumed alcohol (8 g/kg per day) daily for 10 weeks showed that intragastrically feeding them probiotic LGG (2.5 × 10^7^ live once daily) or prebiotic oats (10 g/kg) prevented alcohol-induced GIT dysbiosis (Mutlu et al. 2009). The rats given the interventions had microbiota composition profiles similar to that of control rats that were intragastrically fed an isocaloric dextrose diet for 10 weeks. This finding corresponds to results obtained in an ALD rodent model demonstrating that LGG attenuates endoxtemia and alcoholic steatohepatitis (Nanji et al. 1994). Furthermore, LGG and oat supplementation ameliorates alcohol-induced intestinal oxidative stress, intestinal hyperpermeability, and liver injury in rodent models of alcohol steatohepatitis (Forsyth et al. 2009; Tang et al. 2009). In another study, researchers orally fed C57BL/6 mice the Lieber-DeCarli diet with or without alcohol (5% vol/vol) for 6 weeks and gave a subset of the mice 1 mL of LGG (bacterial density 1 × 10^9^ cfu/mL) orally each day for 6 to 8 weeks (Bull-Otterson et al. 2013). Similar to other findings, the alcohol-fed mice demonstrated a decrease in the abundance of *Bacteriodetes* and *Firmicutes* and an increase in *Proteobacteria* and *Actinobacteria* (table 2). However, probiotic LGG supplementation prevented this alcohol-induced dysbiotic intestinal microbiota composition, especially increasing *Firmicutes*, including *Lactobacillus*. Other studies find that LGG prevents alcohol-induced intestinal hyperpermeability, endotoxemia, and liver injury (Wang et al. 2011, 2013), supporting the notion that LGG may be a therapeutic approach to decrease the development of ALD.

Studies in humans show similar results. One study examined Minimal Hepatic Encephalopathy (MHE) patients with cirrhosis who typically have substantial alterations in their GIT microbiota composition caused by the overgrowth of the potentially pathogenic *Escherichia coli* and *Staphylococcal* species (table 2). Following 30 days of synbiotic and prebiotic treatments, these patients had significantly reduced viable counts of potentially pathogenic GIT microbiota with a concurrent significant increase in fecal content of *Lactobacillus* species (table 2) (Liu et al. 2004). Half of the patients receiving synbiotic treatment also exhibited a significant reduction in blood ammonia levels, endotoxemia, and reversal of MHE, when compared with control subjects. These improvements in MHE correlate with similar findings showing that probiotic supplementation improved hepatic encephalopathy (HE) in patients with cirrhosis (Macbeth et al. 1965). Interestingly, probiotic LGG supplementation prevents alcohol-induced dysbiosis of the intestinal microbial community, and leads to an increase in *Firmicutes,* particularly of the genus *Lactobacillus*. Furthermore, in an U.S. Food and Drug Administration phase I study, the administration of probiotic LGG to cirrhotic patients with MHE (most of whom had Hepatitis C–induced cirrhosis) found that LGG significantly reduces dysbiosis, tumor necrosis factor (TNF)-α, and endotoxemia in comparison to placebo (Bajaj et al. 2014). In addition, LGG shows beneficial changes in the stool microbial profiles and significant changes in metabolite/microbiota correlations associated with amino acid, vitamin, and secondary bile-acid metabolism in comparison to MHE cirrhotic patients randomly assigned to placebo. In a comparison of the synbiotic and prebiotic treatment to cirrhotic patients with MHE in the study above, probiotic LGG does promote beneficial microbiota; however, it does not increase *Lactobacillus* and does not improve cognitive function in the patients for this randomized clinical trial. Thus, taken together, probiotics and/or synbiotics may be a viable approach in humans to alter the GIT microbiota to a more favorable profile to improve clinical outcomes (figure 3).

## Therapeutic Intervention for Treating Alcohol-Induced Intestinal Dysbiosis

The therapeutic intervention studies in this review indicate that in ALD rodent models and MHE alcohol-cirrhosis humans, probiotic and synbiotic intervention increases *Lactobacillus* and *Bifidobacterium* (table 2). These findings suggest that the intestinal microbiota play a role in attenuating alcohol-induced dysbiosis and liver injury. In addition, the modulation of intestinal microbiota could be a viable therapeutic strategy to prevent or normalize alcohol-induced dysbiosis and which would be expected to have beneficial effects on alcohol-induced liver injury as well as other inflammatory-mediated diseases resulting from chronic alcohol consumption.

**Glossary**

Dysbiosis: Dysbiosis is a term used to describe a microbial imbalance on or inside the body, commonly within the digestive tract where it has been associated with illness
Endotoxemia: The presence of endotoxins in the blood, where endoxins are toxic substances bound to the cell wall of certain bacteria
Polymerase Chain Reaction (PCR): A biochemical technology used to amplify a single or a few copies of a particular piece of DNA, generating millions of copies of that DNA sequence. Among other uses, the technique allows researchers to make enough copies of a piece of DNA to sequence it. PCR requires “primers” or small snippets of DNA that match a piece of the DNA researchers are attempting to replicate
Tumor necrosis factor-alpha (TNF-α): A type of cytokine, or cell-signaling protein that can cause cell death

Evidence suggests that probiotic and synbiotic interventions can not only reverse alcohol-induced dysbiosis but can improve the pathogenesis symptoms of the GIT and liver in ALD. Treatment with probiotics prevents or significantly decreases alcohol-induced intestinal permeability (Forsyth et al. 2009; Wang et al. 2012), intestinal oxidative stress and inflammation of the intestine and liver (Forsyth et al. 2009), TNF-α production (Wang et al. 2013), and expression of intestinal trefoil factor and its transcriptional regulator hypoxia-inducible factor-2α (HIF-2α) (Wang et al. 2011) and attenuates endotoxemia and alcoholic steatophepatitis (Nanji et al. 1994) in rodent models and in humans with ALD. Probiotics also restore stool microbiota community structure and liver enzymes in ALD human patients (Kirpich et al. 2008). In addition, prebiotic oat supplementation prevents alcohol-induced gut leakiness in an ALD rat model by preventing alcohol-induced oxidative tissue damage (Tang et al. 2009). Thus, these studies suggest that probiotics (e.g., *Lactobacillus*) transform the intestinal microbiota community composition, which may prevent alcohol-induced dysbiosis, intestinal permeability, bacterial translocation, endotoxemia, and the development of ALD. Transformation of the intestinal microbiota may be a therapeutic target for the treatment of intestinal barrier dysfunction and the development of ALD.

Clinical studies suggest that probiotic consumption of *Lactobacilli, Bifidobacteria,* and *Lactocooci* are effective for the prevention and treatment of a diverse range of disorders (Snydman 2008). History shows that probiotic consumption is safe in healthy people but must still be taken with caution in certain patient groups, including premature neonates, people with immune deficiency, people with short-bowel syndrome, people with central venous catheters, the elderly, and people with cardiac disease (Boyle et al. 2006; Snydman 2008). Clinical trials show that the effects of probiotics are variable depending on age, health, and disease state. Probiotic use also has its concerns. It presents a major risk of sepsis (Boyle et al. 2006) and has been associated with diseases such as bacteremia or endocarditis, toxic or metabolic effects on the GIT, and the transfer of antibiotic resistance in the gastrointestinal flora (Snydman 2008). In addition, the many properties of different probiotic species vary and can be strain specific. Therefore, the effect of new probiotic strains should be carefully analyzed in clinical trials before assuming they are safe to market as a potential therapeutic treatment.

## Future Directions

Chronic alcohol consumption causes intestinal dysbiosis in both rodent models and humans. Dysbiosis in the intestinal microbiota may contribute to the pathogenesis of liver disease by altering intestinal barrier function leading, for example, to gut leakiness, the production of proinflammatory/pathogenic microbial products, and/or liver metabolic pathways. Further investigation into intestinal microbiota composition in alcoholism is necessary to identify new diagnostic as well as therapeutic targets to prevent alcohol-associated diseases, such as ALD. Such therapeutic avenues could include probiotics, prebiotics, synbiotics, or polyphenols to alleviate the symptoms associated with alcohol disorders. Thus, understanding the effect of alcohol on intestinal microbiota composition, may lead to a better understanding of its future functional activity, with the ultimate goal to restore intestinal microbiota homeostasis.

## Figures and Tables

**Figure 1:**
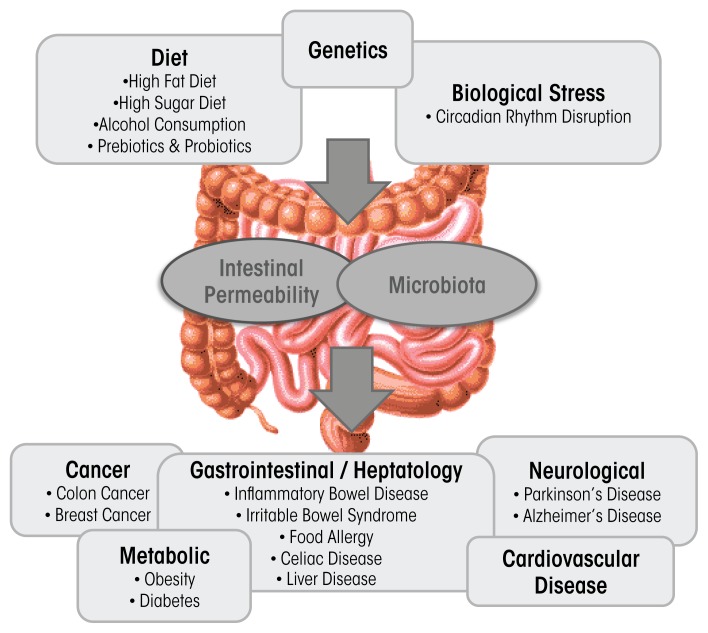
Disruption of intestinal microbiota homeostasis (dysbiosis) has been associated with these diseases (shown above). In addition, dysbiosis can be caused by environmental factors commonly encountered in Western societies, including diet, genetics, disruption of circadian rhythms, and alcoholic beverage consumption. Dysbiosis also can be prevented or treated with probiotics and prebiotics.

**Table 1:** Changes in the Intestinal Microbiome Associated With Alcohol in Rodent Models and Humans

Reference	Tested Organism	Experimental Condition	Methodology	Major Taxa Altered in Presence of Alcohol a,b	Major Finding
Yan et al. 2011	Mouse	3-week alcohol-fed mice/control isocaloric liquid	16S rRNA gene amplicon sequencing (pyro-sequencing) Mouse cecum	↑***Verrucomicrobia*** **phylum:** ↑*Akkermansia* genus ↑***Bacteroidetes*** **phylum:** ↑*Bacteroidetes* class, ↑*Bacteroidales* order, ↑*Bacteroides* genus, ↑*Porphyromonadaceae* family ↓***Firmicutes*** **phylum:** ↓*Lactococcus*, ↓*Pediococcus,* ↓*Lactobacillus*, and ↓*Leuconostoc* genus	Alcohol-fed mice have GIT microbial community composition significantly altered from control mice indicating dysbiosis.
Mutlu et al. 2009	Rat	10-week alcohol-fed rats/control isocaloric dextrose	Length heterogeneity PCR (LH-PCR) Ileal and colonic rat mucosa tissue		Alcohol-fed rats have GIT microbial community composition significantly altered from control rats. Dysbiosis may be an important mechanism of alcohol-induced endotoxemia.
Mutlu et al. 2012	Human	Alcoholics with and without alcoholic liver disease/healthy patients	16S rRNA gene amplicon sequencing (pyro-sequencing) Mucosa sigmoid biopsies	↑***Proteobacteria*** **phylum:** ↑*Gammaproteobacteria* class ***Firmicutes*** **phylum**: ↑*Bacilli* & ↓*Clostridia* class ↓***Bacteroidetes*** **phylum:** ↓*Bacteroidetes* class ***Verrucomicrobia*** **phylum:** ↓*Verrucomicrobiae* class	Human chronic alcohol use is associated with changes in the mucosa-associated colonic bacterial composition in a subset of alcoholics from healthy controls. Dysbiotic microbial community alteration correlated with high level of serum endotoxin.
Chen et al. 2011	Human	Cirrhotic/healthy patients Alcoholic cirrhotic/healthy patients Hepititis B virus cirrhosis/alcoholic cirrhotic patients	16S rRNA gene amplicon sequencing (pyro-sequencing) Fecal samples	↑***Proteobacteria*** **phylum:** ↑*Gammaproteobacteria* class: ↑*Enterobacteriaceae* family ***Firmicutes*** **phylum:** ↑*Bacilli* class: ↑*Streptococcaceae* family; *Clostridia* class: ↑*Veillonellaceae* and ↓*Lachnospiraceae* family ↑***Fusobacteria*** **phylum:** ↑*Fusobacteria* class ↓***Bacteroidetes*** **phylum:** ↓*Bacteroidetes* class *Bacteroidetes **phylum:** ↑*Prevotellaceae* family	Fecal GIT microbial community composition significantly altered in patients with cirrhosis compared with healthy individuals. *Prevotellaceae was enriched in alcoholic cirrhosis patients when compared with HBV cirrhosis patients and healthy controls.
Queipo-Ortuno et al. 2012	Human	Healthy patients 20-day intake of either red wine, de-alcoholized red wine, or gin	Quantitative real-time PCR Fecal samples	**Red wine** ↑***Proteobacteria*** **phylum:** (↓Gin) ↑***Fusobacteria*** **phylum:** (↑De-Alcoholized) (↓Gin) ↑***Firmicutes*** **phylum:** (↓Gin) ↑***Bacteroidetes*** **phylum:** (↓Gin) **Red wine** ↑*Enterococcus* genus (↑De-Alcoholized) (↓Gin) ↑*Prevotella* genus (↑De-Alcoholized) (↓Gin) ↑*Bacteroides* genus (↑De-Alcoholized) (↓Gin) ↑*Bifidobacterium* genus (↑De-Alcoholized) (↓Gin) ↑*Bacteroides* uniformis species: (↑De-Alcoholized) (↓Gin) ↑*Eggerthella lenta* species (↑De-alcoholized) (↓Gin) ↑*Blautia coccoides-Eubacterium rectale* species (↑De-alcoholized) (↓Gin) ↓*Clostridium* genus (↓De-Alcoholized) (↑Gin) ↓*Clostridium histolyticum* species (↓De-alcoholized) (↑Gin)	Red wine consumption, compared to de-alcoholized red wine and gin, significantly altered the growth of select GIT microbiota in healthy patients. This microbial community composition could influence the host’s metabolism. Also, polyphenol consumption suggests possible prebiotic benefits, due to the increase growth of *Bifidobacterium.*
Bode et al. 1984	Human	Alcoholic/hospitalized control patients	Aerobic and anaerobic bacterial culture incubation Jejunum aspirates	↑**Gram-negative anaerobic bacteria** ↑**Endospore-forming rods** ↑**Coliform microorganisms**	Chronic alcohol abuse leads to small intestinal bacterial overgrowth, suggesting dysbiosis may contribute to functional and morphological abnormalities in the GIT.

NOTES:

a A comparison of bacterial Taxa either ↑, increased or ↓, decreased relative to the presence of alcohol.

b Taxonomy was updated using the NCBI Taxonomy Browser.

**Table 2:** Changes in the Intestinal Microbiota Associated With Alcohol and Probiotic or Synbiotic Intervention in Rodent Models and Humans

Reference	Tested Organism	Experimental Condition	Methodology	Major Taxa Altered in Presence of Alcohol a,b	Major Finding
Mutlu et al. 2009	Rat	**10 week:** Control isocaloric dextrose-fed rats/alcohol-fed rats	Length heterogeneity PCR (LH-PCR) Colonic rat mucosa tissue		Alcohol-fed rats have GIT microbial community composition significantly altered from control rats. Both probiotic (LGG) and prebiotic (oats) intervention prevented alcohol-induced dysbiosis, at week 10 in the colonic mucosa tissue of rats.
		**1 week (at week 10):** Alcohol + LGG-fed rats/alcohol + oat-fed rats/dextrose + oat-fed rats			

Bull-Otterson et al. 2013	Mice	**6 week:** Alcohol-fed mice/control isocaloric maltose dextrin-fed mice	16S rRNA gene amplicon sequencing (pyro-sequencing) Fecal mice samples	**Alcohol induced:** ↑***Proteobacteria*** **phylum:** ↑*Alcaligenes* genus ↑***Artinobacteria*** **phylum:** ↑*Corynebacterium* genus ***Firmicutes*:** ↑*Aerococcus,* ↑*Listeria,* ↑*Acetivibrio,* ↑*Clostridiales,* ↑*Allobaculum,* ↑*Lactobacillus* genus	Alcohol-fed mice have fecal GIT microbial community composition significantly altered from control mice. Probiotic (LGG) treatment prevented alcohol induced dysbiosis expansion. LGG reversed the expansion of the *Proteobacteria* and *Actinobaceria* phyla, which could play a pathogenic role in the development of alcoholic liver disease. *Firmicutes* expanded greatly in the alcohol + LGG–fed group.
		**3 week (at weeks 6–8):** Alcohol + LGG-fed mice		>↓***Bacteroidetes*** **phylum:** ↓*Bacteroides,* ↓*Parabacteroides,* ↓*Tannerella,* ↓*Hallella* genus ↓***Firmicutes*** **phylum:** ↓*Lachnospiraceae,* ↓*Ruminococcaceae* genus	
				**Alcohol + LGG:** ↓***Proteobacteria*** **phylum:** ↓*Alcaligene* genus ↓*Artinobacteria* phylum: ↓*Corynebacterium* genus	
				↑***Bacteroidetes*** **phylum** ↑↑↑***Firmicutes*** **phylum:** ↑*Lactobacillus,* ↑*Ruminococcaceae* genus	

Liu et al. 2004	Human	**30-day treatment:** Cirrhotic with MHE + synbiotic or prebiotic or placebo/control patients	Quantitative bacteriological culture Fecal samples	**Cirrhotic with MHE:** ↑ *Escherichia coli* species ↑ *Staphylococcus* genus	Cirrhotic patients with MHE were found to have significant fecal overgrowth of potentially pathogenic gram-negative (*E. coli*) and gram-positive (*Staphylococcus*) aerobic microbiota. After 30 days of synbiotic or prebiotic treatment, supplementation reduced *E. coli, Staphylococcus,* and *Fusobacterium* and increased *Lactobacillus* (Synbiotic) and *Bifidobacterium* (prebiotic) organisms in feces of cirrhotic patients with MHE.
				**Cirrhotic with MHE + synbiotic** ↓ *Escherichia coli* species ↓ *Staphylococcus* genus ↓ *Fusobacterium* genus	
		**Subgroup:** Sober alcoholics 2 weeks & etiology is alcohol-cirrhosis		↑*Lactobacillus* genus	
				**Cirrhotic with MHE + prebiotic** ↓ *Escherichia coli* species ↓ *Fusobacterium* genus	
				↑*Bifidobacterium* genus	

NOTES:

a A comparison of bacterial Taxa either ↑, increased or ↓, decreased relative to the presence of alcohol.

b Taxonomy was updated using the NCBI Taxonomy Browser.
